# Aggressive epithelioid vertebral haemangioma with atypical imaging features presenting as compressive myelopathy: a case series

**DOI:** 10.1093/jscr/rjag135

**Published:** 2026-04-17

**Authors:** Daniela Castaño-Bustos, Mariana Agudelo-Arrieta, María I Ocampo-Navia, Julián A Sierra-Peña, Miguel E Berbeo-Calderón, Roberto C Díaz-Orduz

**Affiliations:** Department of Neurosurgery, Pontificia Universidad Javeriana, Bogotá 110231, Colombia; Department of Neurosurgery, Hospital Universitario San Ignacio, Bogotá 110231, Colombia; Neurosurgery Research Group, Pontificia Universidad Javeriana, Bogotá 110231, Colombia; Department of Neurosurgery, Pontificia Universidad Javeriana, Bogotá 110231, Colombia; Department of Neurosurgery, Hospital Universitario San Ignacio, Bogotá 110231, Colombia; Neurosurgery Research Group, Pontificia Universidad Javeriana, Bogotá 110231, Colombia; Department of Neurosurgery, Pontificia Universidad Javeriana, Bogotá 110231, Colombia; Department of Neurosurgery, Hospital Universitario San Ignacio, Bogotá 110231, Colombia; Neurosurgery Research Group, Pontificia Universidad Javeriana, Bogotá 110231, Colombia; Department of Neurosurgery, Pontificia Universidad Javeriana, Bogotá 110231, Colombia; Department of Neurosurgery, Hospital Universitario San Ignacio, Bogotá 110231, Colombia; Neurosurgery Research Group, Pontificia Universidad Javeriana, Bogotá 110231, Colombia; Department of Neurosurgery, Pontificia Universidad Javeriana, Bogotá 110231, Colombia; Department of Neurosurgery, Hospital Universitario San Ignacio, Bogotá 110231, Colombia; Department of Neurosurgery, Pontificia Universidad Javeriana, Bogotá 110231, Colombia; Department of Neurosurgery, Hospital Universitario San Ignacio, Bogotá 110231, Colombia

**Keywords:** embolization, epithelioid hemangioma, magnetic resonance imaging, spinal cord compression, thoracic vertebrae

## Abstract

We report two cases of atypical vertebral haemangiomas. The first case involves a 44-year-old male presenting thoracolumbar pain, abdominal paresthesias, a T7 sensory level, and paraparesis. Imaging revealed an osteolytic lesion of the T3 vertebral body, epidural extension and spinal cord compression. An initial corpectomy with stabilization was performed, and histopathology was consistent with haemangioma. After partial neurological recovery, the patient experienced deterioration due to tumour regrowth. Preoperative embolization followed by combined posterior and thoracotomy-assisted resection allowed extended tumour removal and spinal stabilization, with sustained improvement at 12 months follow-up. The second case concerns a 65-year-old female with lumbar pain radiating to the left L5 dermatome and mild paresis. Magnetic resonance imaging demonstrated an L5 vertebral body lesion hypointense and hyperintense on T1/T2-weighted sequences. Biopsy confirmed diagnosis. These are benign lesions; however, atypical or aggressive forms—1%—may mimic malignancy leading to neurological compromise. This highlights the importance of early diagnosis and multidisciplinary management.

## Introduction

Vertebral hemangiomas (VH) are among the most common benign spinal tumors, with an estimated prevalence of 10%–12% in the general population [1, 2]. Histologically, these vascular lesions are classified into capillary, cavernous, venous, or mixed subtypes, with capillary and cavernous variants being the most frequently encountered [3]. Histological classification is essential to differentiate VH from other vascular or neoplastic spinal lesions.

From a clinical and radiological perspective, VH are categorized as typical, atypical, or aggressive [4, 5]. Typical lesions are usually incidental findings and remain asymptomatic. Atypical hemangiomas, accounting for ~1%–2% of cases, exhibit unusual imaging characteristics that frequently mimic malignancy, posing a significant diagnostic challenge [6–9]. Aggressive hemangiomas represent roughly 1% of cases and may extend into the spinal canal or posterior elements, resulting in pain, radiculopathy, or myelopathy [4, 10].

Atypical and aggressive hemangiomas often lack classic imaging features such as the ‘polka-dot’ sign on computed tomography (CT) or hyperintensity on both T1- and T2-weighted magnetic resonance imaging (MRI) sequences [2], thereby resembling metastatic disease, plasmacytoma, or lymphoma [4, 11]. Consequently, these lesions require a broad differential diagnosis and careful diagnostic evaluation.

We present a case series of two patients. The first case describes a rapidly progressive aggressive epithelioid hemangioma of the thoracic spine with atypical imaging features and epidural extension. Its rare histology and malignant-like behaviour underscore the importance of early recognition and timely intervention. The second case involves a patient with lumbar pain and L5 radiculopathy caused by an atypical vertebral hemangioma currently undergoing treatment. The inclusion of an ongoing case is intentional and educational, as it illustrates the early diagnostic process, histopathological confirmation, and initial therapeutic planning in atypical vertebral hemangiomas. Given the heterogeneous behaviour and unpredictable clinical course of aggressive VH, early-stage cases provide valuable insights despite the absence of long-term follow-up, which is acknowledged as a limitation [1, 8, 12, 13]. This further emphasizes the importance of early diagnosis and management.

## Case 1

A 44-year-old male presented with a 20-day history of abdominal paresthesias and axial thoracolumbar pain, without preceding trauma. Ten days after symptom onset, he developed left foot drop, progressive contralateral weakness, and a defined sensory level. On admission, neurological examination revealed proximal muscle strength of 2/5 and distal strength of 1/5 in the left lower limb, and 4+/5 proximally and 5/5 distally in the right lower limb, in the MRC/Daniels strength scale. A T7 sensory level, hyperreflexia, and bilateral Babinski signs were present.

**Figure 1:**
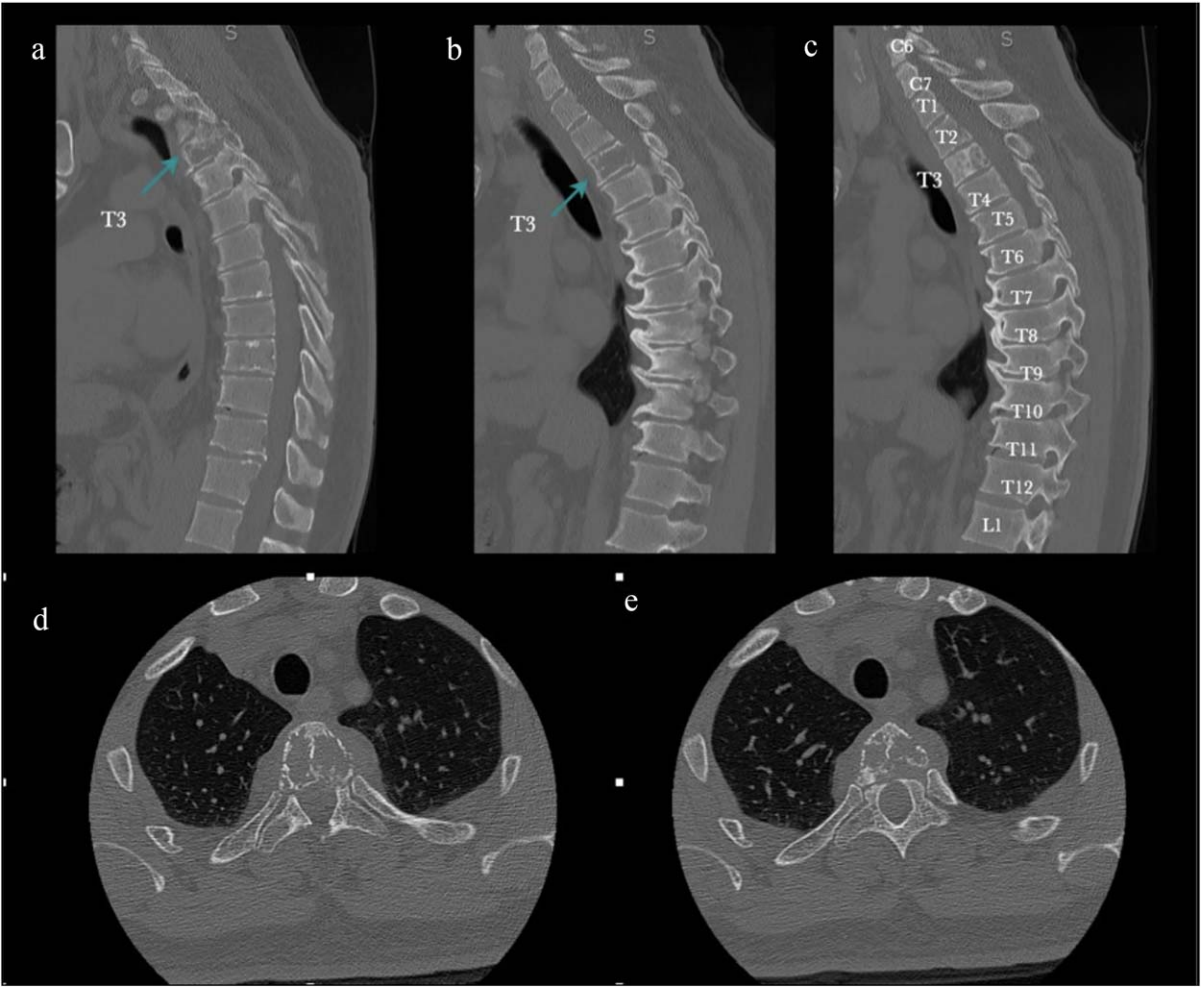
Preoperative CT. (a–c) Sagittal bone window: Osteolytic T3 vertebral body lesion. (d, e) Axial views: Vertebral body compromise. The arrow shows the T3 level.

**Figure 2:**
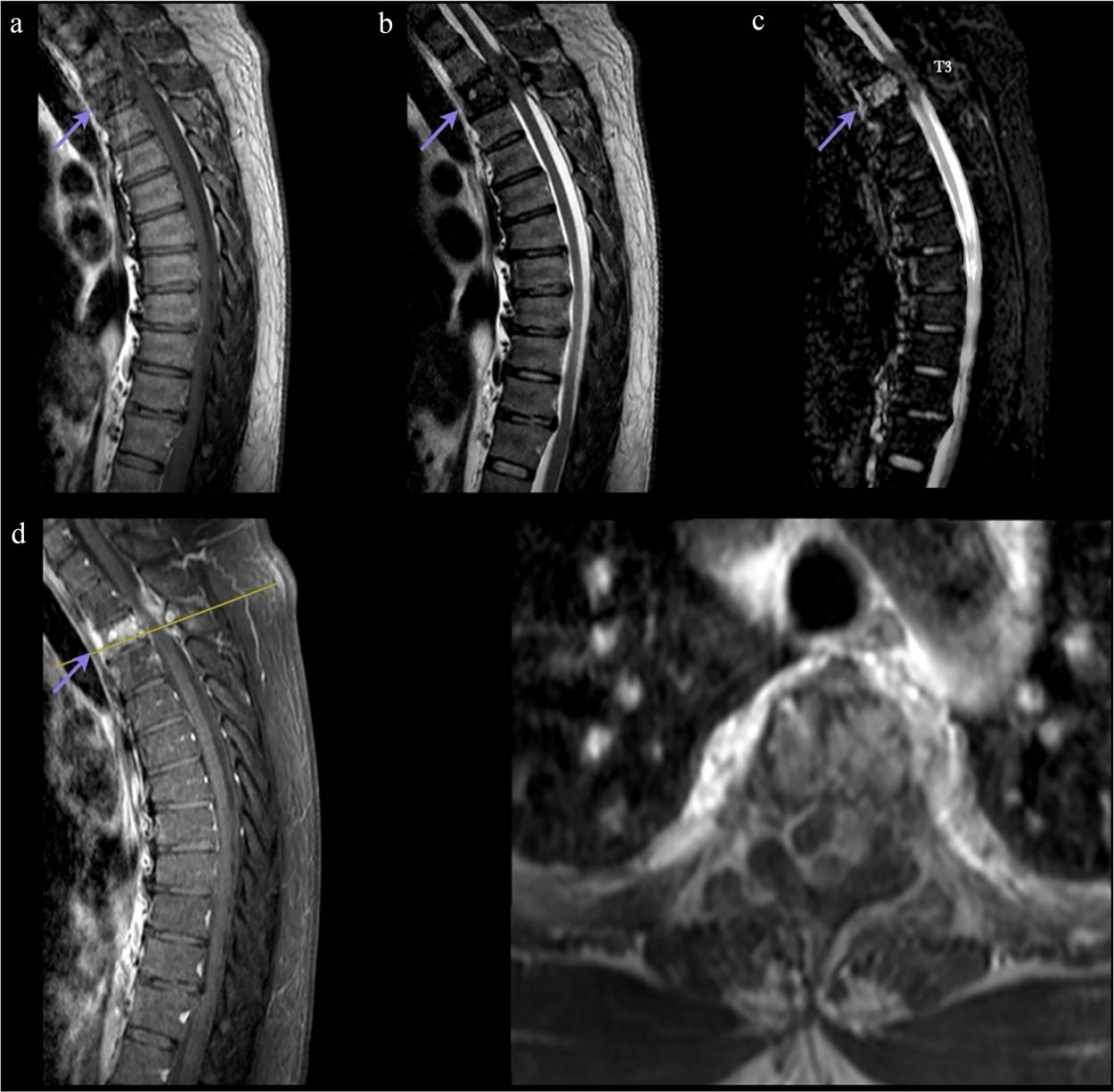
Preoperative MRI. (a, b) T1/T2 sagittal: Hypointense T3 lesion with epidural extension and cord compression. (c) STIR sagittal: Hyperintensity with myelopathic changes (T2–T4). (d) Post-gadolinium sagittal/axial: Contrast enhancement. The line indicates the T3 level. Arrow marks T3 level.

MRI and CT demonstrated an infiltrative osteolytic lesion involving the T3 vertebral body with posterior and lateral epidural extension, resulting in severe spinal cord compression. The lesion appeared hypointense on T1- and T2-weighted sequences, hyperintense on STIR, and showed heterogeneous gadolinium enhancement. Associated spinal cord signal changes extended from T2 to T4 (Figs 1 and 2).

The differential diagnosis included metastatic disease and aggressive vertebral hemangioma. Systemic corticosteroid therapy resulted in partial neurological improvement. Staging studies excluded systemic malignancy.

**Figure 3:**
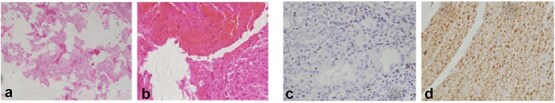
Histopathology. (a, b) Hematoxylin and eosin (H&E) staining showing vascular proliferation with epithelioid endothelial cells and fibrous stroma. (c) Smooth muscle actin (SMA) immunostaining highlights vascular walls. (d) Nuclear positivity for an ETS-related gene (ERG) confirms endothelial origin. Findings are consistent with epithelioid hemangioma.

Given progressive neurological deficits and spinal instability, the patient underwent a left T3 laminectomy and partial corpectomy with cage placement and posterior instrumentation from T2 to T5. Intraoperatively, the lesion was noted to be highly vascular. Histopathological analysis confirmed the diagnosis of epithelioid hemangioma (Fig. 3).

Postoperatively, the patient demonstrated neurological improvement with recovery of strength and ambulation. However, 4 weeks later, he developed recurrent gait impairment, thoracic sensory loss, neuropathic pain in the T2–T5 dermatomes, and progressive motor deterioration, now involving the right lower limb. He also developed cervical kyphosis with inability to maintain primary gaze.

Follow-up imaging revealed residual tumour growth with extension into the right paravertebral and coastal regions from T3 to T5, associated with foraminal stenosis. Spinal angiography demonstrated vascular supply from the right T4 intercostal artery and the thyrocervical trunk.

**Figure 4:**
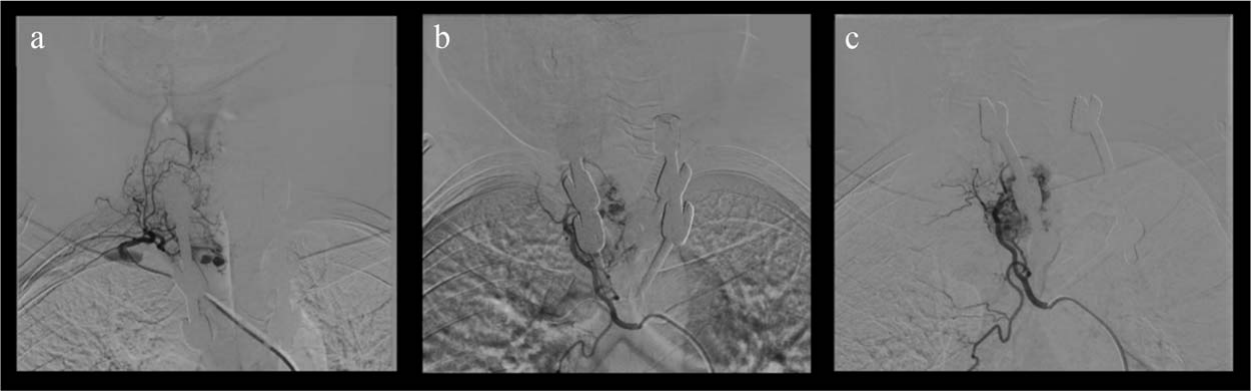
Preoperative spinal angiography. (a) Selective injection: Hypervascular T3 lesion supplied by intercostal branches. (b, c) Post-embolization: Devascularization achieved.

Due to clinical deterioration, preoperative embolization followed by reoperation was performed (Fig. 4). A thoracoscopic-assisted thoracotomy combined with a posterior approach provided wide exposure and tumor control. After pleural cavity entry, the third and fourth ribs were resected with ligation of the intercostal neurovascular bundles. Dissection of the costotransverse joints from T2 to T4 allowed direct access to the lesion. The previously placed right rod was temporarily removed to improve visualization. Epidural scar tissue and recurrent friable vascular tumor were carefully dissected using microsurgical technique with adjunctive hemostatic measures. Circumferential decompression of the dural sac was achieved. A dural tear occurred and was repaired primarily with sutures and fibrin sealant. The rod was subsequently repositioned and secured, restoring posterior instrumentation and spinal stability.

**Figure 5:**
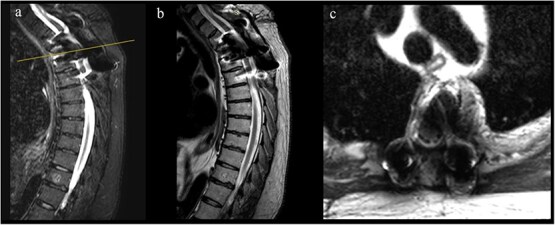
1-year follow-up MRI. (a, b) Sagittal T2-weighted images showing decompression of the spinal cord at T3 with resolution of the epidural component and susceptibility artifact from arthrodesis material. (c) Axial T2-weighted image confirming adequate canal decompression with artifact related to instrumentation.

Postoperatively, neurological recovery was significant. Left lower limb strength improved to 4+/5 with complete resolution of foot drop, and right lower limb strength returned to normal. Mild residual weakness, spasticity, and a persistent T7 sensory level remained, but neuropathic pain resolved. At 12-month follow-up, the patient demonstrated sustained functional improvement with no radiological evidence of recurrence (Fig. 5). He continues multidisciplinary follow-up and rehabilitation.

## Case 2

A 65-year-old female presented with a one-year history of progressive lumbar pain radiating to the left lower limb, described as an electric shock–like sensation, associated with subjective weakness. Her medical history included treated thyroid cancer in remission, hypothyroidism, and hypertension.

Neurological examination demonstrated full strength in the upper limbs and right lower limb. In the left lower limb, strength was reduced to 4+/5 in knee extension and hallux dorsiflexion. Sensory examination was normal, and generalized hyperreflexia was observed.

**Figure 6:**
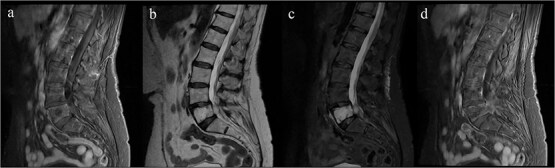
Preoperative MRI showing a L5 vertebral body lesion with extension to the left foramina and radicular obliteration. (a) Sagittal T1- weighted sequence with hypointense signal. (b) T2 and STIR hyperintense signal. (c) Minimum gadolinium enhancement.

**Figure 7:**
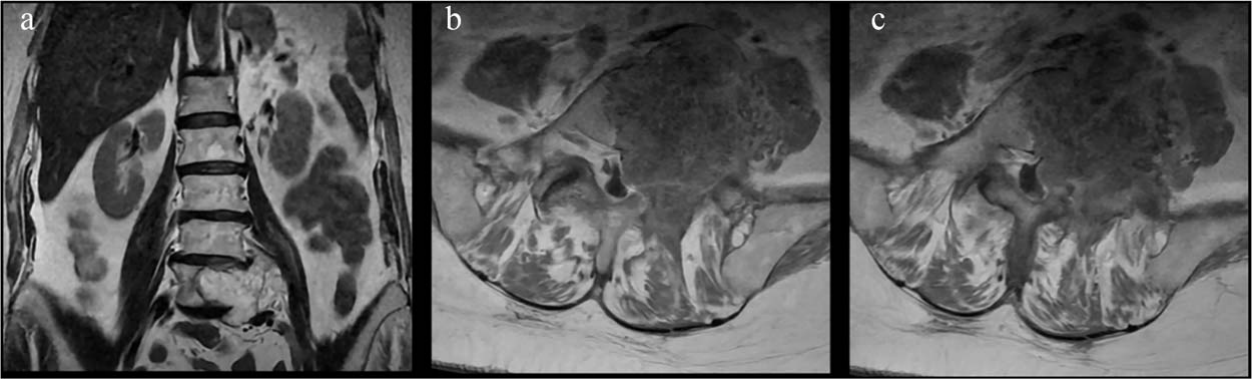
Coronal and axial views show an expansile L5 vertebral body lesion with heterogeneous T2 hyperintensity and internal trabeculation, consistent with vertebral hemangioma, which occupies most of the vertebral body with partial involvement of the pedicles and left foramina.

Imaging revealed a heterogeneous lesion of the L5 vertebral body with extension into the left L5 neural foramen, causing radicular compression. The lesion was hypointense on T1-weighted images and hyperintense on T2-weighted and STIR sequences, with minimal gadolinium enhancement (Figs 6 and 7).

The patient underwent CT-guided biopsy and a selective left L5 nerve root block, which provided transient symptom relief lasting 2 days. Histopathological examination with immunohistochemistry demonstrated CD34 positivity in the vascular component, negative staining for other markers, and a near-zero Ki-67 proliferation index. These findings supported a benign vascular lesion consistent with vertebral hemangioma.

Based on these results, surgical treatment was indicated. The patient is scheduled for preoperative embolization followed by tumor resection with L5 corpectomy and posterior instrumented fusion from L4 to the iliac bones using a dual-rod construct to maintain spinal stability and sagittal balance.

## Discussion

Spinal hemangiomas account for approximately 28% of skeletal hemangiomas and are most frequently located in the thoracic spine, with reported prevalence rates of up to 11% in autopsy series [4, 14, 15]. They are more common in women aged 40–60 years and may be multiple in up to one-third of patients.

Although most VH remain asymptomatic, ~1% becomes symptomatic, typically corresponding to aggressive subtypes [1, 7, 8]. Atypical hemangiomas lack classic imaging features and frequently mimic malignant tumours, complicating diagnosis [4, 5, 7–9]. Aggressive lesions demonstrate expansile growth, cortical erosion, and epidural extension, leading to pain, radiculopathy, or myelopathy [4, 10, 12, 13].

Our cases differ from previously reported aggressive VH by demonstrating a combination of atypical imaging, rapid neurological deterioration, early postoperative recurrence, and confirmation of epithelioid histology. While many aggressive hemangiomas follow a relatively indolent course after decompression, the first case exhibited malignant-like behavior despite benign histology, necessitating reintervention with preoperative embolization and a combined surgical approach. The second case illustrates an earlier stage of atypical VH with radiculopathy and imaging features mimicking metastatic disease, highlighting the diagnostic value of percutaneous biopsy and early therapeutic planning before neurological decompensation.

Histologically, hemangiomas are classified as capillary, cavernous, venous, or mixed, with rare aggressive variants including epithelioid hemangioma and hemangioendothelioma [4, 12, 16]. Epithelioid hemangioma is characterized by well-formed vascular channels lined by epithelioid endothelial cells with minimal atypia and low mitotic activity, distinguishing it from epithelioid hemangioendothelioma and angiosarcoma [16, 17]. Immunohistochemical positivity for endothelial markers such as CD34 and ERG, combined with a low Ki-67 index, supports a benign or low-grade biological behavior and has direct implications for prognosis and treatment planning [3, 15, 16].

Radiologically, typical VH demonstrate the classic polka-dot or corduroy signs on CT and hyperintensity on T1- and T2-weighted MRI [2]. Atypical and aggressive lesions deviate from this pattern and closely resemble metastatic disease [4, 5]. Advanced MRI techniques have been explored, but biopsy remains the gold standard for definitive diagnosis [5, 18, 19].

Management must be individualized. Surgical decompression is the mainstay in symptomatic patients, with embolization, vertebroplasty, and radiotherapy serving as adjunctive therapies [4, 5, 14, 20]. Preoperative embolization is particularly valuable in highly vascular lesions, reducing intraoperative blood loss and facilitating safer resection. Multimodal treatment strategies may be required in complex cases to achieve optimal outcomes [5, 9–12, 20].

## Conclusion

VH are common and usually benign; however, atypical and aggressive forms may closely mimic malignant disease and result in significant neurological compromise. The epithelioid variant, although exceedingly rare, underscores the critical role of histopathological confirmation. Symptomatic aggressive lesions often require surgical intervention complemented by embolization or other multimodal strategies. Early recognition, accurate diagnosis, and multidisciplinary management are essential to achieve favorable clinical outcomes.
